# A copper-controlled RNA interference system for reversible silencing of target genes in *Trichoderma reesei*

**DOI:** 10.1186/s13068-018-1038-7

**Published:** 2018-02-09

**Authors:** Lei Wang, Fanglin Zheng, Weixin Zhang, Yaohua Zhong, Guanjun Chen, Xiangfeng Meng, Weifeng Liu

**Affiliations:** State Key Laboratory of Microbial Technology, School of Life Science, Shandong University, No.27 Shanda South Road, Jinan, 250100 Shandong People’s Republic of China

**Keywords:** RNAi, Gene function, *Trichoderma reesei*, Copper-responsive promoter

## Abstract

**Background:**

*Trichoderma reesei* is a primary lignocellulosic enzyme producer in industry. However, the mechanisms underlying cellulase synthesis as well as other physiological processes are insufficiently understood partly due to the sophisticated process for its genetic manipulation. Target gene knockdown by RNA interference (RNAi) is a powerful tool for genetic research and biotechnology in eukaryotes including filamentous fungi. Previously reported RNAi system in *T. reesei* was either uncontrollable or only applicable in certain nutrition state.

**Results:**

In the present study, we incorporated the copper-responsive *tcu1* promoter into an RNAi-mediated silencing system to develop a controllable RNAi-mediated silencing system in *T. reesei*. As the proof-of-concept, a prototrophic *pyr4* gene, highly expressed *cel7a* and *xyr1* genes induced by Avicel and a *fab1* gene, whose knockout has proved to be intractable, were successfully knocked down in the absence of copper when the respective RNAi fragment was expressed. Importantly, the phenotype of RNAi strains was shown to be reversed easily to mimic the complementation for excluding any unwanted effects resulted from the random integration of the hpRNA cassette by adding copper in the media. Thus, this controllable RNAi-mediated silencing system can be turned on and turned off only depending on the absence and presence of copper ions in the media, respectively, and not on the nutritional states.

**Conclusions:**

The copper-controlled RNA interference system represents an effective tool for reversible silencing of target genes in *T. reesei.* This reported strategy to conditionally knock down or turn off genes will contribute to our understanding of *T. reesei* gene functions, especially those that are difficult to be knocked out due to various reasons. In addition, this simple and cost-effective method holds great potential for the application in synthetic biology and genetic engineering of *T. reesei.*

**Electronic supplementary material:**

The online version of this article (10.1186/s13068-018-1038-7) contains supplementary material, which is available to authorized users.

## Background

Bioconversion of the abundant renewable plant biomass consisting of lignocellulose holds great potential for the production of environmental friendly energy and chemicals [1]. Lignocellulose is primarily made of various interconnected biopolymers (e.g., cellulose, hemicellulose and lignin) and displays significant structural heterogeneity and complexity, which render them recalcitrant to degradation [2]. The industrial use of plant biomass involves mechanical, chemical or physicochemical pretreatment steps followed by enzymatic hydrolysis to release the constituent monosaccharides for further fermentation to produce biofuels and biochemicals [3, 4]. As for the enzymatic hydrolysis step, multiple lignocellulosic enzymes, including cellulases, hemicellulases and polysaccharide lyases, are required to act synergistically to depolymerize cellulose and hemicellulose [5–7]. Preparation and use of these enzymes are therefore a major cost factor either due to the low catalytic performance and/or to the suboptimal composition of the enzyme cocktail under industrial conditions [8, 9].

The large amounts of lignocellulosic enzymes (e.g., cellulases and hemicellulases) secreted by the ascomycete *Trichoderma reesei* represent the prominent source of enzyme cocktails that are used in industry for biomass degradation [10–12]. Although industrial *T. reesei* strains have been reported to produce cellulases over 100 g/L [13], the cost of enzyme production used in the saccharification step is still considered to be the major bottleneck of biomass conversion for the economical production of biofuels and biochemicals. Not surprisingly, *T. reesei* has been the subject of intensive investigation toward the improvement of its cellulases including increasing or reinforcing their existing activity and production to reduce the costs for biofuel production. The production of lignocellulosic enzymes by *T. reesei* involves many physiological processes including extracellular signal sensing [14, 15], intracellular signal transduction [16], transcriptional regulation of cellulase genes and protein synthesis [17, 18], and secretion [19]. Understanding the molecular mechanism behind these processes is crucial to further improve cellulase productivity of *T. reesei*.

Although the genomic sequence of *T. reesei* is now available, genes without an explicit function make up a large portion of the genome due to limited and intricate genetic manipulation approaches [20]. The gene knockout strategy by homologous recombination (HR) has been developed and widely used in *T. reesei* to study the function of particular genes [21, 22]. However, HR in *T. reesei* usually involves labor-intensive plasmid constructions for incorporating sufficiently long homologous arms on both sides of the target gene to ensure its occurrence at an inherent low efficiency [23]. As a result, large amounts of screening efforts are then needed to obtain the appropriate knockout strain. Although the low efficiency of HR has been partially solved by inactivating the genes (e.g., *tku*70) in non-homologous end joining (NHEJ) pathway [23, 24], deleting these important DNA repair genes may exert unanticipated side effects. To solve this issue, Chum et al. [25] recently improved the efficiency of HR to certain extent by only transiently silencing *tmus53* gene of NHEJ pathway. Nevertheless, the gene knockout strategy is improper to study a large proportion of important or essential genes, whose deletion proves to be intractable and results in severe growth defect or even inviability, making the phenotypic characterization impossible. On the other hand, tunable promoters controlled by external conditions have been shown to be useful for investigating the function of target genes in *T. reesei* [26, 27]. Developing effective genetic manipulation systems by combining these induced or repressible promoters with conventional gene-targeting strategies is thus of prior importance to fully characterize such essential genes and genes with unknown functions.

The post-transcriptional gene silencing by small interfering RNA (siRNA)-mediated RNAi is triggered by double-stranded RNA and exists extensively in eukaryotes [28]. Soon after its discovery, RNAi becomes a powerful tool for functional genomic study due to its targeted nature, silencing of genes to the equivalent of null mutants and applicability to any transformable species of lower or higher eukaryotes [29, 30]. In many cases, RNAi is initiated by expressing a target gene fragment cloned as a tandem inverted repeat separated by a hairpin-forming intron sequence [29, 31]. The transcript creates a hairpin RNA (hpRNA) that then serves as a template for the RNAi machinery. There have been several studies describing the modulation of transcript abundance of specific genes in *T. reesei* using RNAi [31, 32]. Specifically, Qin et al. [31] have reported an inducible RNAi system with the *cel7a* promoter allowing induction of hpRNA expression in response to cellulose. Schmoll et al. [32] reported the knocking down of *gan3*, an adenylate cyclase-activating class III of G-alpha subunit, by expressing its antisense RNA driven by constitutive *gpd1* promoter in *T. reesei*. These systems, however, were either uncontrolled or relied on the transition of nutritional state which may result in many other side effects complicating the explanation for the observed phenotypes.

Here, we present a copper-controlled RNAi-mediated knockdown system in *T. reesei* by combining the previously identified copper-responsive P_*tcu1*_ promoter with the routine RNAi vector for reversible silencing of different target genes. The usefulness of this system was demonstrated by the successful down-regulation of target genes in response to the absence of exogenous copper ions while maintaining their normal expression in the presence of copper. This copper-controlled RNAi system provides an alternative approach to characterize the functions of genes in *T. reesei*, especially those genes whose expression is under stringent regulation and thus extremely sensitive to any fluctuation in its expression level.

## Methods

### Strains and culture conditions

*Escherichia coli* DH5α was used for plasmid construction and cultured in lysogeny broth with a rotary shaker (200 rpm) at 37 °C. QM9414 (ATCC 26921) and a uridine auxotroph of *T. reesei* TU-6 (ATCC MYA-256) were used in this work as parental strains. The *pyr4* gene-deleted strain of QM9414 (QM9414–∆*pyr4*) was used as a control strain for *pyr4* gene knocking down. All *T. reesei* strains were maintained on malt extract agar or liquid media supplemented with 10 mM uridine when necessary. For the transcription and cellulase production analysis, spores were pre-grown in 1-L Erlenmeyer flasks on a rotary shaker (200 rpm) at 30 °C with 250 mL Mandels–Andreotti (MA) media containing 1% (v/v) glycerol as the carbon source for 48 h as previously described [18]. Mycelia were harvested by filtration and washed twice with media without a carbon source. Equal amounts of mycelia were then transferred to fresh media without peptone containing 1% (w/v) Avicel and incubation was continued for the indicated time periods. Copper was present or absent perpetually in the media from pre-culture to induction. For vegetative growth on agar plates, equal amounts of growing mycelia or spores were inoculated on minimal media agar plates containing 1% (w/v) glucose at 30 °C for several days. The Avicel double-layer plates where the minimal media agar without carbon source was covered by 1% (w/v) Avicel were used for investigating cellulase production.

### Construction of plasmids and strains

**Fig. 1:**
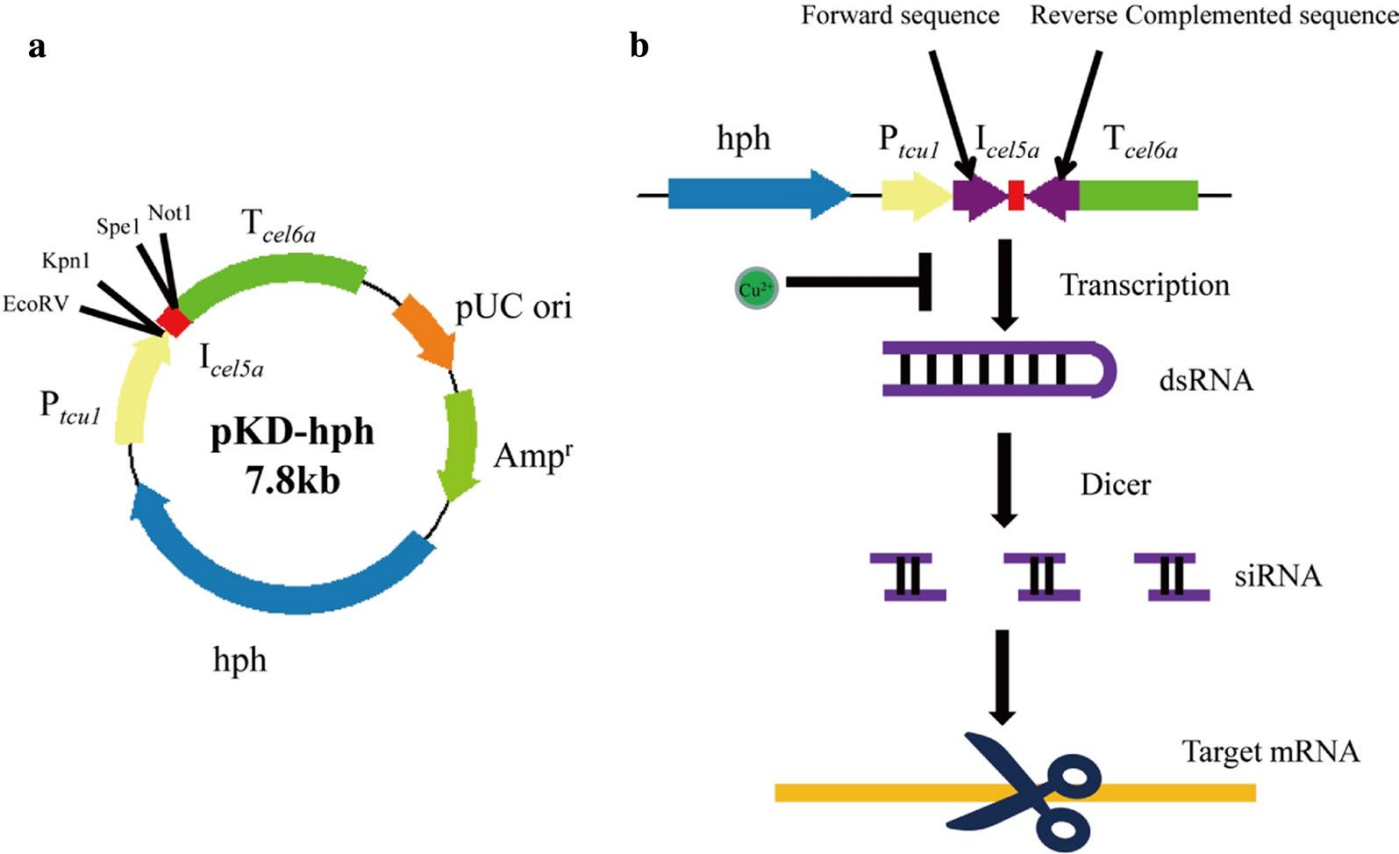
Map of pKD-*hph* vector showing the main features and restriction sites used for plasmids construction. P_*tcu1*_ and T_*cel6a*_ were used as promoter and terminator, respectively, for the expression of RNAi fragments. A *cel5a* intron was inserted between P_*tcu1*_ and T_*cel6a*_ (**a**). Two reverse complemented RNAi fragments were inserted into the pKD-*hph* vector using *Eco*RV–*Kpn*1 and *Spe*1–*Not*1, respectively. The transcribed hpRNA fragments driven by the P_*tcu1*_ promoter can be processed by Dicer into siRNA, which then directs degradation of the target gene mRNA by RNAi mechanism. The transcription of RNAi fragment can be turned off when copper was present in the media (**b**)

The 744-bp *Hin*dIII–*Eco*RV fragment containing P_*tcu1*_ promoter, 1041-bp *Spe*1–*Sal*1 fragment containing T_*cel6a*_ terminator and 179-bp *Eco*RV–*Spe*1 fragment containing I_*cel5a*_ intron were amplified by PCR from the genomic DNA of *T. reesei* QM9414 and then inserted into pMD19T-*hph* vector containing the hygromycin-resistance gene by an orderly manner (Fig. 1a) for the construction of pKD-*hph* vector. Restriction sites *Eco*RV–*Kpn*1 and *Spe*1–*Not*1 are used to insert forward and reverse complemented RNAi fragment, respectively. The 788-bp *Eco*RV–*Kpn*1 fragment containing *xyr1* cDNA sequence and reverse complemented *Spe*1–*Not*1 fragment were amplified by PCR from the total cDNA of Avicel-inducing *T. reesei* QM9414 and then were ligated into pKD–*hph* (Fig. 1b), generating pKD-*hph*–*xyr1*. The 905-bp *cel7a* cDNA sequence, 736-bp *pyr4* cDNA sequences and 714-bp *fab1* cDNA sequences, and their reverse complemented fragments, were inserted into pKD-*hph* in the same way as pKD-*hph*–*xyr1*, resulting in pKD-*hph*–*cel7a*, pKD-*hph*–*pyr4* and pKD-*hph*–*fab1*, respectively. To construct P_*tcu1*_–*xyr1*^KD^ strain, the linearized pKD-*hph*–*xyr1* vector with *Ssp*1 was used to transform *T. reesei* strain. Transformation of *T. reesei* was carried out essentially as described by Penttila [21]. Transformants were selected on minimal media containing 120 μg/mL hygromycin. Genomic PCR was used to verify correct integration events using a 2×T5 Super PCR Mix (TSE005, Beijing Tsingke Biotech Co., Ltd) as described in Additional file 1. Primers for plasmids construction and genomic PCR are also provided in Additional file 1. The knocking down of target genes was confirmed by comparing their mRNAs level in the media supplied with and without CuSO_4_.

### Enzyme activity and protein analysis

The activities of cellobiohydrolases (pNPCase) and β-glucosidases (pNPGase) were determined by measuring the amount of released *p*-nitrophenol using *p*-nitrophenyl-β-d-cellobioside (pNPC; Sigma, St. Louis, United States) and *p*-nitrophenyl-β-d-glucopyranoside (pNPG; Sigma) as substrates, respectively. The pNPC and pNPG activity assays were performed in 200 μL reaction mixtures containing 50 μL of culture supernatant and 50 μL of the respective substrate plus 100 μL of 50 mM sodium acetate buffer (pH 4.8) and then incubated at 45 °C for 30 min. One unit (U) of pNPCase or pNPGase activity was defined as the conversion of 1 μmol of substrate per minute under the test conditions as described previously [33]. The endo-glucanases and the filter paper activities (FPA) were determined by measuring the released reducing sugar with carboxymethylcellulose sodium salt (CMC; Sigma) and filter paper as substrates, respectively. Determination of the CMC hydrolytic activities was carried out at 50 °C in a 100 μL reaction mixture containing 50 μL of appropriately diluted culture supernatant and 50 μL of 0.5% (w/v) CMC sodium in 50 mM sodium acetate buffer (pH 4.8). The FPA assay was performed at 50 °C in a 200 μL reaction mixture including 50 μL of appropriately diluted culture supernatant and 150 μL 50 mM sodium acetate buffer (pH 4.8) with Whatman No. 1 filter paper as substrates. One unit (U) of CMCase or FPA was defined as the release of 1 μmol reducing sugar per minute under the test conditions [33]. Total secreted and intracellular proteins were determined using the Bradford protein assay with bovine serum albumin (BSA) as a standard. SDS-PAGE and Western blotting were performed according to the standard protocols and Cel7a was immunoblotted using a polyclonal antibody raised against amino acids 426–446 of the protein as previously described [34].

### Quantitative RT-PCR (qRT-PCR)

Total RNAs were extracted using the Trizol reagent (Invitrogen, Grand Island NY, United States) and purified using the TURBO DNA-free kit (Ambion, Austin TX, United States) to eliminate the genomic DNA contamination according to the manufacturer’s instructions. Reverse transcription was performed using the PrimeScript RT reagent Kit (Takara, Japan) according to the instructions. Quantitative PCR was performed using SYBR green supermix (TaKaRa, Japan) on a Bio-Rad myIQ2 thermocycler (Bio-Rad, California, United States). Data were analyzed using the relative quantitation/comparative threshold cycle (ΔΔCT) method and were normalized to the endogenous gene actin. Primers for qRT-PCR are provided in Additional file 1. Three biological replicates were performed for each analysis and the results and errors are the mean and standard deviation (SD), respectively. Statistical analysis was performed using the Student’s *t* test analysis.

### Designed culture procedure for investigating the *fab1* gene function in *T. reesei* using copper-responsive RNAi system

In order to exclude the indirect effects of growth difference brought from pre-culture, we took P_*tcu1*_–*fab1*^KD^ strain as an example to redesign a culture strategy to characterize the function of *fab1* in cellulase production in *T. reesei.* First, copper was added into MA media containing 1% glycerol as a sole carbon source in the first pre-culture for 36 h. Harvested mycelia were washed five times with media without a carbon source and then transferred to fresh MA media supplied without copper. After 12 h culturing, mycelia were harvested again and washed two times with media without a carbon source. Finally, mycelia were transferred to induced media containing 1% Avicel supplied without copper. As a control, P_*tcu1*_–*fab1*^KD^ strain was also culture in parallel with copper perpetually in the media to repress the expression of *fab1* gene RNAi fragments. The mRNA level of *tcu1* and *fab1* were analyzed in the ending of first (Fig. 6b, c: sampling time S1) and second (Fig. 6b, c: sampling time S2) pre-culture by qRT-PCR. Besides, their mRNA abundance also was analyzed after mycelia grow under induced condition at 6 h (Fig. 6b, c: sampling time S3) and 12 h (Fig. 6b, c: sampling time S4), respectively. Cellulase production was assessed at 12, 24, 36, 48 60 and 72 h after the mycelia were transferred to the Avicel inducing media by measuring the FPA activity and SDS-PAGE analysis.

## Results

### Design of copper-responsive RNAi-mediated silencing system

The controllability of the P_*tcu1*_ promoter was incorporated into an RNAi-mediated silencing system in *T. reesei*. As shown in Fig. 1, gene-specific sequence was cloned as a tandem inverted repeat arranged on both sides of an intron from the *cel5a* gene to build synthetic hpRNA fragments, which was then inserted downstream of the P_*tcu1*_ promoter. The transcription of hpRNA fragments for target genes will result in the formation of double-stranded RNA that is recognized by Dicer and then produces siRNA to induce degradation of target mRNAs. In our present strategy, hpRNA fragments driven by P_*tcu1*_ are thus expected to down-regulate the transcript levels of the target gene when no copper ions were included in the media. Silencing can be reversed by adding back copper ions to mimic gene complementation.

### Copper-responsive RNAi-mediated silencing of the prototrophic gene *pyr4*

**Fig. 2:**
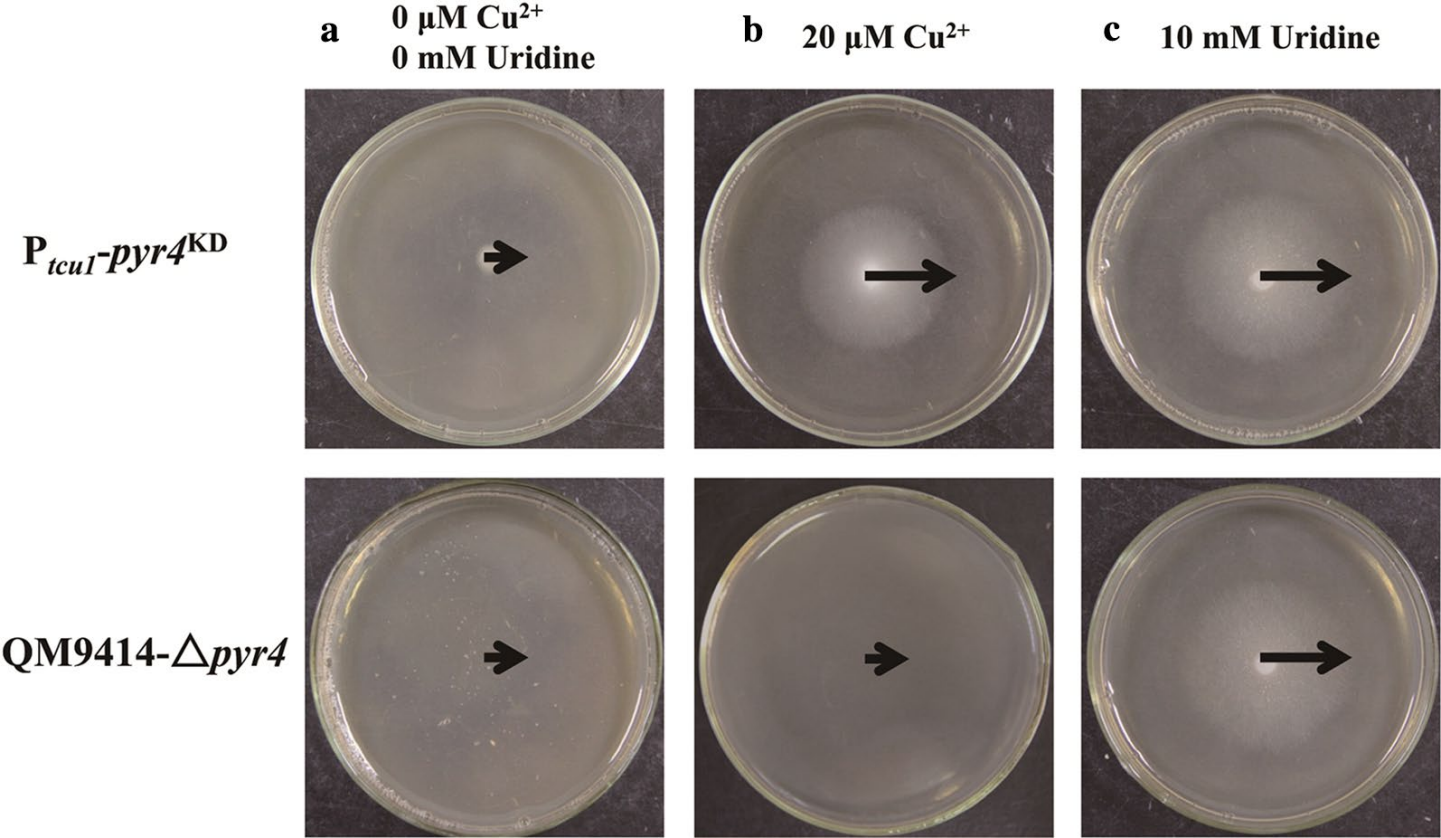
Knockdown of *pyr4* using P_*tcu1*_-driven RNAi system was responsive to copper in agar plate. Equal amount of conidia spores from QM9414 and the P_*tcu1*_–*pyr4*^KD^ strain was inoculated on minimal media agar plate containing 2% (w/v) glucose as a sole carbon source. **a** No copper and uridine was present in the media. Twenty μM copper (**b**) and 10 mM uridine (**c**) were added into the minimal media agar plates, respectively

The *pyr4* gene encodes an orotidine 5′-phosphate decarboxylase which is involved in the uridine biosynthesis. Knockout of endogenous gene *pyr4* in *T. reesei* showed that cells cannot survive unless uridine is present in the media [35]. *Pyr4* is thus widely used as a screening marker in the genetic manipulation of *T. reesei* [22]. In order to test whether the RNAi-mediated silencing system can efficiently work in response to exogenous copper, the *pyr4-*specific hpRNA fragments were inserted downstream of the P_*tcu1*_ promoter in the pKD-*hph* vector, generating the pKD-*hph*–*pyr4* plasmid. Transformants (P_*tcu1*_–*pyr4*^KD^) were first selected on minimal media for resistance to hygromycin. Hyphal growth of selected P_*tcu1*_–*pyr4*^KD^ strains on minimal media agar plates with glucose as the sole carbon source was investigated. As showed in Fig. 2a, P_*tcu1*_–*pyr4*^KD^ spores barely germinated in glucose minimal media agar plate without addition of copper, which is also observed with the control strain QM9414–∆*pyr4*, demonstrating that the *pyr4* gene was knocked down by the RNAi-mediated silencing system. Adding 20 μM copper (Fig. 2b) or 10 mM uridine (Fig. 2c) in the media rescued the growth, equivalent to the restored growth of QM9414–∆*pyr4* on media only with 10 mM uridine (Fig. 2c). Under the above-tested conditions, the growth of QM9414–∆*pyr4* was hardly affected by copper. These results revealed that the P_*tcu1*_ promoter controlled the transcription of *pry4* RNAi fragment to knock down of *pyr4* gene in response to the external copper.

### Copper-controlled RNAi-mediated knockdown of cellulose-induced genes

**Fig. 3:**
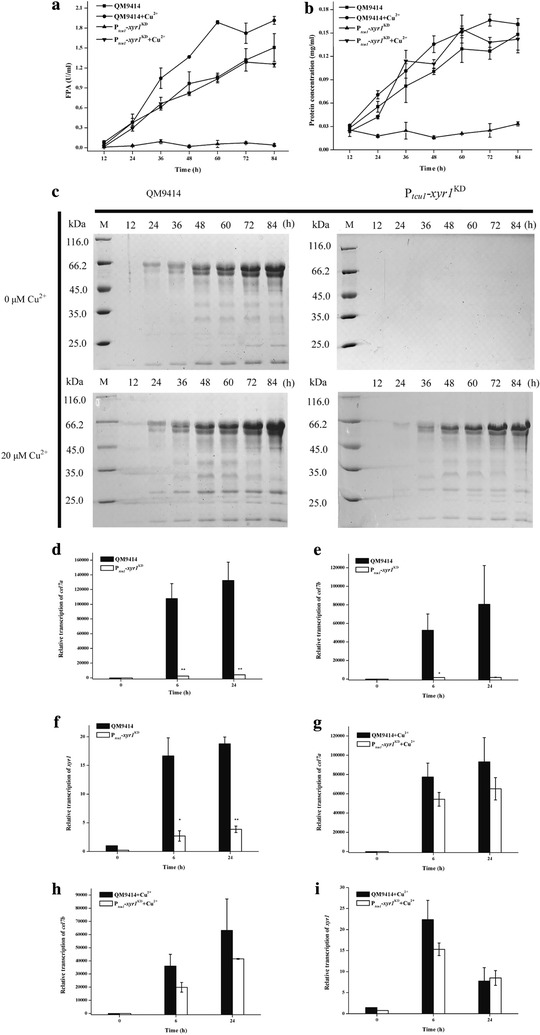
The effect of *xyr1* knockdown by copper-responsive RNAi-mediated silencing system. The extracellular FPA (**a**) and protein concentration (**b**) of the parental and P_*tcu1*_–*xyr1*^KD^ strains cultured on 1% (w/v) Avicel supplied with or without CuSO_4_ were determined as described in “Methods” section. SDS-PAGE analysis of the total extracellular protein accumulation (**c**) of the P_*tcu1*_–*xyr1*^KD^ and the parental strains cultured on 1% (w/v) Avicel supplied with or without CuSO_4_, respectively, at the indicated time points. qRT-PCR analysis of intracellular mRNA levels of the endogenous *cel7a*, *cel7b*, *and xyr1* in parental and P_*tcu1*_–*xyr1*^KD^ strains was performed at the indicated time points with (**g**–**i**) or without (**d**–**f**) CuSO_4_. A significant difference (*t* test *P* < 0.05) was detected for the expression of *cel7a*, *cel7b*, and *xyr1* in parental and P_*tcu1*_–*xyr1*^KD^ strains. Error bars are SD from three biological replicates

To further test the effectiveness of the developed system, we selected two cellulose-induced genes for our proof-of-concept study. XYR1 is a critical activator for the transcription of cellulase and hemicellulase genes in *T. reesei*, deletion of which resulted in the abolishment of the induced production of nearly all (hemi)cellulases [36, 37]. The transcription of *xyr1* itself has been also shown to be induced by cellulosic substrates such as Avicel and by lactose in *T. reesei* [38]. CEL7A is the major cellulase component produced by *T. reesei* and is shown to be tightly regulated by XYR1 [39, 40]. *T. reesei* QM9414 was first transformed with the pKD-*hph*–*xyr1* and the cellulolytic activity of selected RNAi transformant was determined. As shown in Fig. 3a, the FPA was almost abolished in the P_*tcu1*_–*xyr1*^KD^ strain just as that of the ∆*xyr1* strain, but remained at a similar level to that of QM9414 strain when 20 μM copper was present in the Avicel inducing media. In contrast to the P_*tcu1*_–*xyr1*^KD^ strain, only a slight increase of the extracellular FPA was observed with copper compared to that without copper for QM9414. The extracellular protein concentration of the P_*tcu1*_–*xyr1*^KD^ strain was also found to be dramatically reduced in the absence of copper, but maintained a similar level to that of the parental strain QM9414 in the presence of copper. SDS-PAGE analysis verified that the overall secreted proteins of the P_*tcu1*_–*xyr1*^KD^ strain under inducing conditions were severely reduced without copper, whereas no significant difference was observed with QM9414 when induced in the presence or absence of copper (Fig. 3c). To test whether the reduced FPA and extracellular protein were the results of down-regulation of *xyr1* mRNAs, we determined the transcript level of *xyr1* and that of cellulase genes *cel7a* and *cel7b* by quantitative qRT-PCR. The abundance of *xyr1* mRNAs in the P_*tcu1*_–*xyr1*^KD^ strain was obviously reduced compared to that of QM9414 under Avicel inducing conditions without copper (Fig. 3f). In contrast, *xyr1* mRNAs in the P_*tcu1*_–*xyr1*^KD^ strain were restored to a similar level to that QM9414 when copper ions were added to the Avicel inducing media (Fig. 3i). Similar to the case of ∆*xyr1*, the transcription of *cel7a* and *cel7b* in the P_*tcu1*_–*xyr1*^KD^ strain was severely reduced upon induction in the absence of copper (Fig. 3d, e). Adding 20 μM copper ions into the inducing media restored their transcription to levels comparable to those of the QM9414 strain (Fig. 3g, h).

**Fig. 4:**
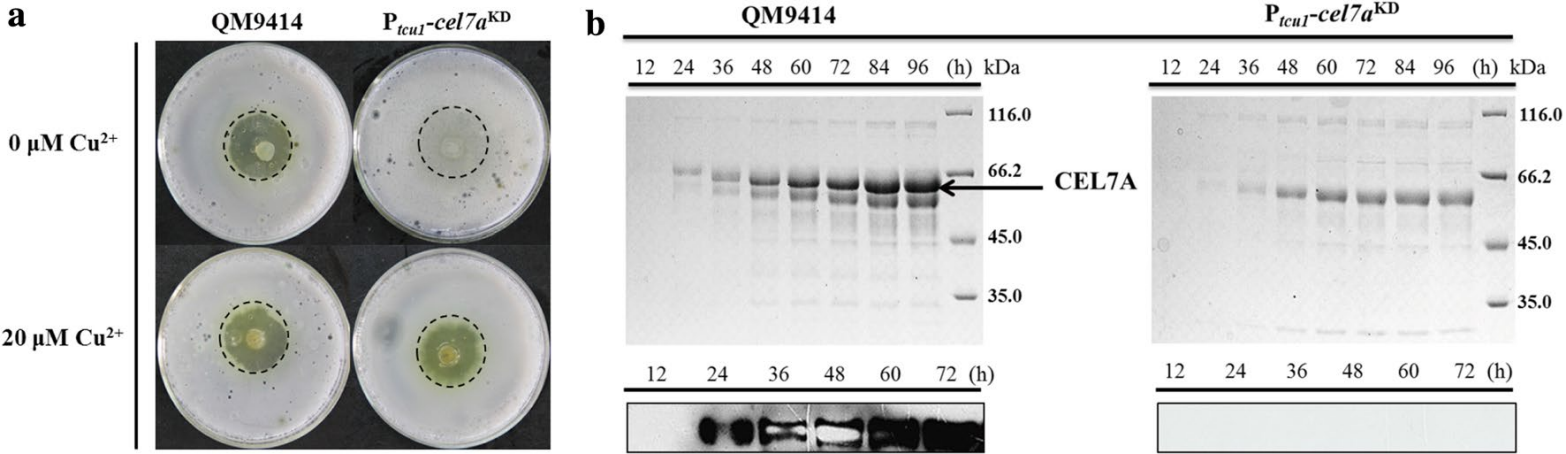
Knockdown of *cel7a* by the copper-responsive RNAi-mediated silencing system. Mycelial growth on double-layer agar plate containing 1% (w/v) Avicel supplied with or without copper (**a**). The parental and the P_*tcu1*_–*xyr1*^KD^ strains were cultured in Avicel-induced media without copper. The total extracellular proteins from both strains were analyzed by SDS-PAGE and Western blot at the indicated time points (**b**)

Copper-controlled RNAi-mediated down-regulation of the highly transcribed *cel7a* was also tested. As shown in Fig. 4a, the parental strain QM9414 showed apparent hydrolytic halo on Avicel both with and without 20 μM copper. Similar hydrolytic halo was observed for the RNAi strain when 20 μM copper was included in the media. However, the P_*tcu1*_–*cel7a*^KD^ strain only displayed a very limited hydrolysis of Avicel when no copper was added. SDS-PAGE and Western blot analysis demonstrated that CEL7A was not detected in the extracellular supernatant of the P_*tcu1*_–*cel7a*^KD^ strain in the absence of copper upon induction (Fig. 4b). Together, these results demonstrated that highly expressed *xyr1* and *cel7a* induced by Avicel could be successfully knocked down by the P_*tcu1*_-driven RNAi approach in a controllable manner wherein the expression of XYR1 and CEL7A became responsive to the external copper levels in the media.

### Copper-controlled silencing of *fab1* encoding phosphatidylinositol 3,5-bisphosphate kinase compromises cellulase production

**Fig. 5:**
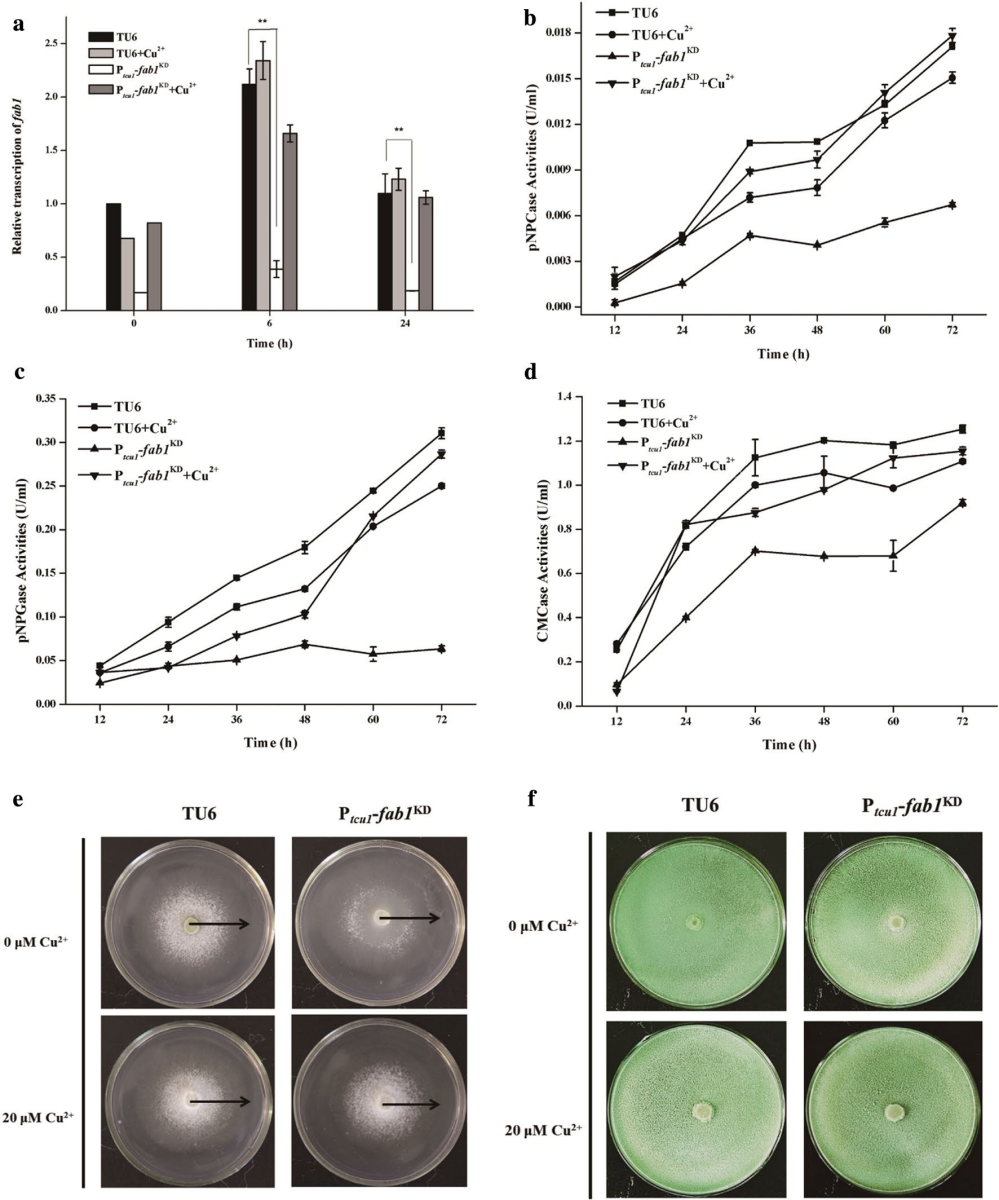
Knockdown of *fab1* by copper-responsive RNAi-mediated silencing system. The abundance of *fab1* mRNAs (**a**), extracellular pNPC (**b**), pNPG (**c**) and CMC (**d**) hydrolytic activity of the parental and P_*tcu1*_–*fab1*^KD^ strains cultured on 1% (w/v) Avicel supplied with or without copper were determined. Mycelial growth (**e**) and sporulation (**f**) of the parental TU6 and P_*tcu1*_–*fab1*^KD^ strains on agar plate containing 1% glucose and malt extract agar plate, respectively, supplied with or without copper were investigated

*Trichoderma reesei fab1* gene (Tr_78274) is annotated to encode phosphatidylinositol-3-phosphate 5-kinase homolog catalyzing the phosphorylation of phosphatidylinositol 3-phosphate (PtdIns(3)P) to yield phosphatidylinositol 3,5-bisphosphate (PtdIns(3,5)P_2_) [41, 42]. Deletion of *fab1* in *Saccharomyces cerevisiae* has been reported to result in severe growth defect and exhibited enlarged vacuole probably due to the defect in vacuolar membrane recycling/turnover [43, 44]. Moreover, PtdIns(3,5)P_2_ has been reported to play a role in recruiting SAGA complex to the *gal1* gene promoter to activate the gene transcription in *S. cerevisiae* [45]. We thus made multiple attempts to delete the *fab1* homolog in *T. reesei* by HR for investigating its function on cellulase production but without success. This failure implied that *fab1* may be essential for viability because we did not obtain strains with a complete removal of the endogenous *fab1* gene (data not shown). Thus, we chose to conditionally knock down the expression of *fab1* in *T. reesei* using the copper-responsive RNAi silencing strategy. As shown in Fig. 5a, the P_*tcu1*_–*fab1*^KD^ strain showed an approximately 80% decline in *fab1* transcript abundance in the absence of copper upon Avicel induction, whereas no significant effect was observed when the RNAi strain was cultured with 20 μM copper. In addition, RNAi-mediated down-regulation of the *fab1* gene did not result in a visible phenotype regarding mycelial growth and sporulation (Fig. 5e, f). To further evaluate the function of *fab1* in cellulase induction, pNPCase, pNPGase and CMCase activities of the parental strain and the P_*tcu1*_–*fab1*^KD^ strain cultured on 1% (w/v) Avicel with or without copper were determined (Fig. 5b–d). The results revealed that the parental strain showed similar pNPCase, pNPGase and CMCase activity upon induction regardless of the presence or absence of copper, whereas the P_*tcu1*_–*fab1*^KD^ strain displayed significantly decreased hydrolytic activities in the absence of copper. Supplementing copper to the inducing media restored the production of the above cellulases in the RNAi strain. Interestingly, the observed defect in cellulase induction in the P_*tcu1*_–*fab1*^KD^ strain did not seem to occur at the transcription level since there was no significant reduction in transcript abundance of the *cel7a* and *cel7b* genes when cultured without copper (Additional file 1).

**Fig. 6:**
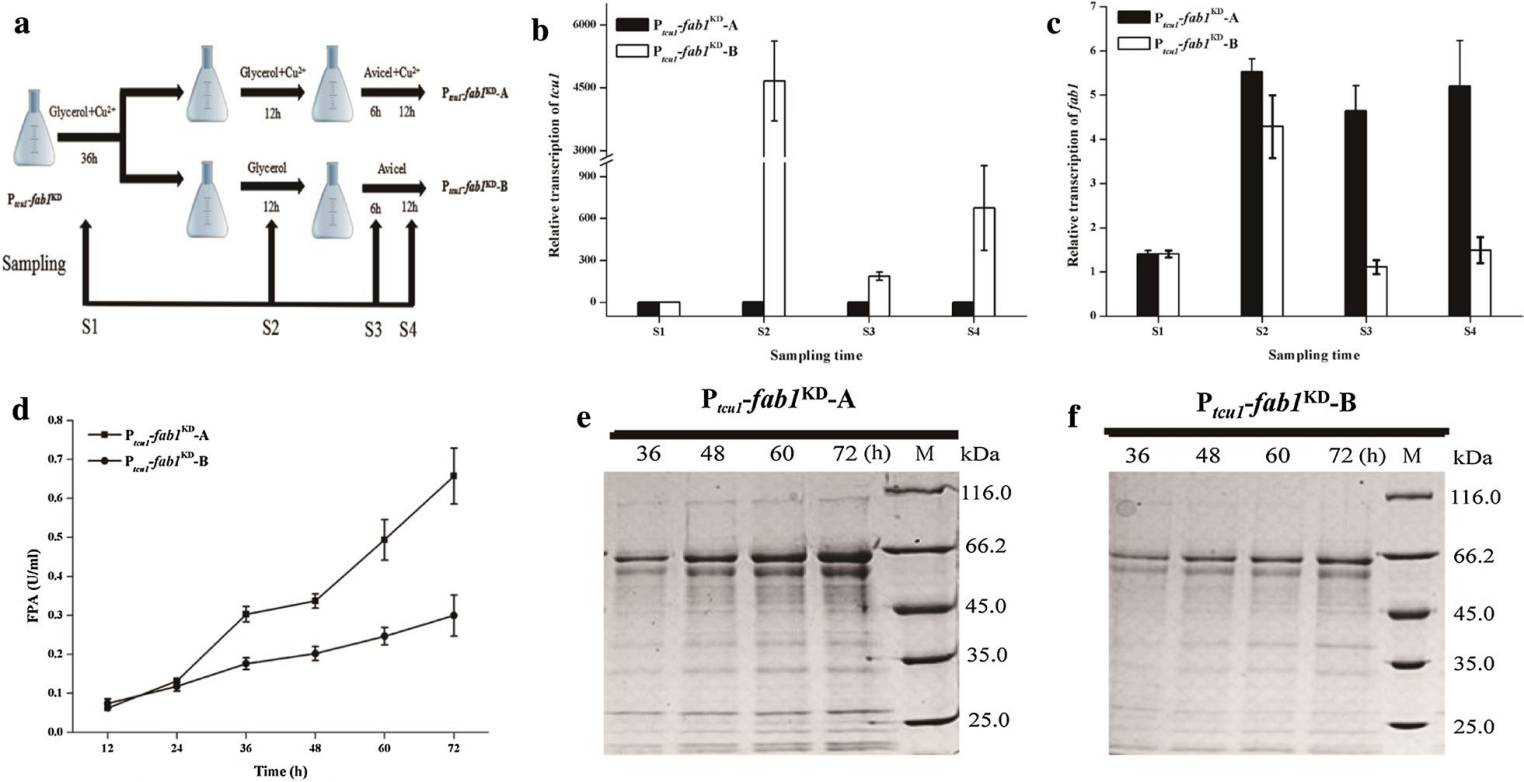
Redesigned culture strategy to verify the functional role of *fab1* in cellulase production. Two culture procedures (**a**) were designed for functional study of *fab1* in *T. reesei* using the copper-responsive RNAi-mediated silencing system. The abundance of *fab1* (**b**) and *tcu1* (**c**) mRNAs was quantified by qRT-PCR in the appointed sampling times. Filter paper activity was measured when mycelia were transferred into Avicel induction media at indicated time (**d**). Extracellular fermentation liquor (20 μL) of P_*tcu1*_*–fab1*^KD^ strain cultured on 1% Avicel supplied with (**e**) or without (**f**) copper was analyzed by SDS-PAGE

In order to exclude any indirect effect by growth difference originating from the pre-culture on phenotype characterization, we also took the P_*tcu1*_–*fab1*^KD^ strain as an example to compare different culture strategies to verify the role of *fab1* in cellulase production in *T. reesei.* As shown in Fig. 6a, the P_*tcu1*_–*fab1*^KD^ strain was pre-cultured for 36 h on glycerol with 20 μM copper. The transcription of the *fab1* hpRNA fragment was repressed during this process with the P_*tcu1*_–*fab1*^KD^ strain displaying the same growth as the parental strain. After extensive washing, the pre-cultured mycelia were inoculated in fresh glycerol media with or without copper and incubated for 12 h, allowing the siRNAs targeting the *fab1* mRNA to be accumulated during this period only in the absence of copper. Mycelia after this stage of growth were finally transferred into Avicel media with and without copper, respectively. The transcript abundance of *tcu1* and *fab1*, filter paper activity, and extracellular protein concentrations of culture samples taken at specific time points (Fig. 6a: sampling time S1, S2, S3, S4) during the whole process were determined. As expected, the *tcu1* promoter was immediately switched on when the pre-cultured mycelia were transferred to glycerol media without copper and maintained a relatively high-level expression after being transferred to the corresponding inducing conditions (Fig. 6b: sampling time S2). The transcript level of *fab1* was, however, only slightly down-regulated at the sampling time S2 compared with that in the presence of copper (Fig. 6c: sampling time S2), indicating that this short period of culture before cellulase induction would contribute to the initiation of the RNAi process toward the target gene. After 6- (Fig. 6c: sampling time S3) and 12-h (Fig. 6c: sampling time S4) induction on Avicel, the abundance of *fab1* mRNA reduced dramatically in the absence of copper, which was in sharp contrast with the no obvious decrease in *fab1* mRNA quantity in the presence of copper. Correspondingly, the P_*tcu1*_–*fab1*^KD^ strain displayed significantly reduced extracellular FPA and protein accumulation upon induction without copper compared those with copper (Fig. 6d–f).

## Discussion

*Trichoderma reesei* is capable of secreting a large amount of proteins and represents a major workhorse for production of cellulases and other recombinant proteins as well [46]. Since its first isolation and the discovery for its astonishing potential to produce extracellular cellulases, extensive efforts have been made to further improve the production of *T. reesei* cellulases and thus to facilitate its industrial application through classical mutagenesis and screening, culminating in the isolation of strain RUT-C30 with a malfunction of the catabolite repressor CRE1 [47]. It is noteworthy that some strains used for the production of cellulases and hemicellulases on the industrial scale are descendants of RUT-C30 [48]. With the advent of fungal gene manipulation techniques, molecular biological strategies including HR-based gene deletion or overexpression were increasingly employed for strain improvement [49]. Such attempts have been best demonstrated by the introduction of β-glucosidases as well as other accessory proteins into *T. reesei* to act in synergistic way with *T. reesei* cellulases during biomass hydrolysis [5, 48, 50]. It is therefore not surprising that modern commercial cellulase preparations are usually based on genetically modified strains of *T. reesei* possessing rather high β-glucosidase activity [51]. In the genomic and post-genomic era, comparative genomic studies of *T. reesei* mutant strains resulted in the identification of potentially critical factors in lignocellulosic enzyme production and regulation, which further facilitate the rational design and construction of lignocellulosic enzyme hyper-production strains and the optimization of the enzyme profiles of *T. reesei* by genetic engineering [52]. This, however, requires a more versatile molecular tool box that enables easy manipulation of genes which is a prerequisite for strain engineering.

Although homologous recombination has been the major approach for producing knockout strains in *T. reesei* with varying levels of success, RNAi as a powerful tool in the functional genomic studies in eukaryotes could be employed for partial or condition-specific suppression of gene expression, particularly when gene deletion could be detrimental or even lethal. An ideal inducible RNAi system should be inactive in the un-induced state to prevent undesired gene silencing, and be rapidly induced to be able to repress target gene expression and also be reversible. The ability to manipulate gene expression in this way is thus anticipated to facilitate dissecting the functions of genes that give lethal or complex pleiotropic phenotypes in knockout mutants or stable RNAi transformant. In this study, we successfully knocked down the constitutively expressed *pyr4* gene, Avicel inducing *xyr1* and *cel7a* genes using the developed copper-controlled RNAi system. The RNAi strains displayed the same phenotype as that of deletion strains when copper was absent in the media by down-regulating the target gene expression, but behaved similarly as the parental strains when copper was present in the media. These data thus indicated that the developed responsive RNAi system could on the one hand readily knock down/off the target gene allowing the phenotypic characterization, whereas on the other hand it could mimic the complementation to exclude any unwanted effects resulted from the random integration of the hpRNA cassette by adding copper ions in the media. Compared with previously developed RNAi system with the constitutive or inducible promoter in *T. reesei* [31, 32], the present copper-responsive RNAi system does not depend on certain nutrition state and thus is applicable in any culture media. Importantly, the conditional silencing of the target gene allows the possibility to exclude any effect exerted by the differential growth between the RNAi strain and the control strain by specifically designed culture strategies. It should be pointed out that it would be convenient if the copper-controlled RNAi system could be turned on by directly adding specific copper chelators to the culture. Although our previous study demonstrated that trace amount of copper ions in the culture (< 200 nM) had hardly any effect on the expression of the *tcu1* gene, the copper chelator bathocuproinedisulfonic acid (BCS) could only partially relieved the *tcu1* repression and may therefore not be ideal for this system [28]. Nevertheless, we found that directly adding the copper to the culture can shut down the expression of hpRNA, simply washing the culture before transfer to a fresh medium without copper is an effective way to turn on hpRNA expression.

Taking advantage of the controllability of our copper-responsive RNAi-mediated gene silencing system, we investigated the function of *fab1* gene for cellulase production, whose deletion was proven to be intractable with repetitive attempts. Our results showed that the knocking down of *fab1* significantly reduced the cellulase production, whereas transcriptional analysis revealed that cellulase gene transcription was not affected. Thus, we speculated that RNAi-mediated down-regulation of the *fab1* gene may interfere with other aspects of cellulase gene expression including protein sorting and transport rather than transcription. Considering that RNAi-mediated down-regulation of the *fab1* gene showed no visible phenotypic deficiency regarding mycelial growth and sporulation, the failure to delete *fab1* by homologous recombination was probably not due to the growth defect of the resultant mutant strain. Nevertheless, possibility cannot be ruled out that a low level of *fab1* transcripts due to the incomplete silencing of *fab1* was sufficient for the growth of the P_*tcu1*_–*fab1*^KD^ strain while the complete knockout of *fab1* was lethal. The *fab1* gene may thus play differential roles in *T. reesei* and the exact mechanism of *fab1* on cellulose production requires further studies.

Previously, we developed a promoter replacement system, in which P_*tcu1*_ promoter was used to substitute the target endogenous promoter in situ [53]. Including cooper in the media results in the repression of target gene transcription while overexpression of target genes is achieved by excluding the copper in the media. The usefulness of this system has been illustrated by investigating the function of two subunits (Gcn5 and Ada2) of the putative Spt–Ada–Gcn5 acetyltransferase complex, whose knockouts were proven to cause severely defect in mycelia growth and spore formation [53, 54]. However, we noticed that the repression of some genes transcribed at a low level by P_*tcu1*_ promoter replacement system was not successful (data not shown), probably due to the slight leakage of the P_*tcu1*_ promoter. On the other hand, in the absence of copper, the high-level expression of the target genes in the promoter-replaced strain would sometimes complicate the phenotypic analysis. In the present study, we further developed a P_*tcu1*_-driven RNAi-mediated silencing system to solve this issue. Thus, combining the P_*tcu1*_ promoter replacement system and the present P_*tcu1*_-driven RNAi-mediated silencing system provides us the effective complementary tool set for the overexpression and knocking down of most genes in *T. reesei* for functional genomic studies.

## Conclusion

In this study, we developed an RNAi-mediated silencing system driven by the P_*tcu1*_ promoter which is highly responsive to the copper ions. The developed RNAi system could readily knock down/off the target gene in the absence of copper allowing the phenotypical characterization and could mimic the complementation of the deficient strain simply by including copper in the media to exclude the unwanted effect that may result from the random integration of the hpRNA cassette. The copper-responsive RNAi-mediated silencing system is applicable on different nutritional states and represents a powerful tool for characterizing target gene functions in *T. reesei*.

## Additional file

**Additional file 1.** Figures and Table.

## Abbreviations

P_*tcu1*_: *tcu1* promoter
RNAi: RNA interference
HR: homologous recombination
NHEJ: non-homologous end joining
siRNA: small interfering RNA
hpRNA: hairpin RNA
FPA: filter paper activities
CMC: carboxymethylcellulose sodium salt
pNPC: *p*-nitrophenyl-β-d-cellobioside
pNPG: *p*-nitrophenyl-β-d-glucopyranoside
PtdIns(3)P: phosphatidylinositol 3-phosphate
PtdIns(3,5)P_2_: phosphatidylinositol 3,5-bisphosphate
BCS: bathocuproinedisulfonic acid
