# Supplementary material for: A copper-controlled RNA interference system for reversible silencing of target genes in Trichoderma reesei
Source: Biotechnol Biofuels. 2018 Feb 9;11:33. doi: 10.1186/s13068-018-1038-7 (PMC5806297; doi:10.1186/s13068-018-1038-7)
Supplement: Supplementary file 1 — Additional file 1. Figures and Table. [file 13068_2018_1038_MOESM1_ESM.pdf]

Additional file

A copper controlled RNA interference system for reversible silencing of target genes in *Trichoderma reesei*

Lei Wang, Fanglin Zheng, Weixin Zhang, Yaohua Zhong, Guanjun Chen, Xiangfeng Meng*, Weifeng Liu

No.27 Shanda South Road, State Key Laboratory of Microbial Technology, School of Life Science, Shandong University, Jinan 250100, Shandong, P. R. China

Corresponding to Xiangfeng Meng; email: x.meng@sdu.edu.cn.

Genomic PCR for verifying correct integration event

The correct integration of RNAi fragment cassette in the chromosome was verified by genomic PCR. We designed four universal primers to amplified two parts of RNAi fragment cassette using primer pairs P_{tcu1}-F(F1)/I_{cel5a}-R(R1) and I_{cel5}-F(F2)/T_{cel6a}-R(R2) (Figure S1). The PCR result showed that two 1000 bp DNA fragments were produced using F1/R1 and F2/R2 primers, respectively, using P_{tcu1}-*pyr4*^{KD} strain genome as a template (Figure S2A), which were consistent with expectation (1018 bp and 999 bp, respectively). Likewise, 1070 bp and 1051 bp PCR products were obtained using genome of P_{tcu1}-*xyr1*^{KD} strain as a template (Figure S2B), 1187 bp and 1168 bp PCR productions were amplified using genome of P_{tcu1}-*cel7a*^{KD} strain as a template (Figure S2C) and 996 bp and 977 bp DNA fragments were produced using genome of P_{tcu1}-*fab1*^{KD} strain as a template (Figure S2D).

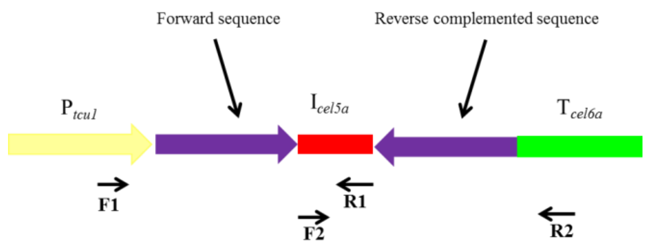

Figure S1 Four universal primers were used for verifying correct integration events of RNAi fragment cassette. The pairs of primers P_{tcu1}-F(F1)/I_{cel5a}-R(R1) and I_{cel5}-F(F2)/T_{cel6a}-R(R2) were used for verifying forward sequence and reverse complement sequence of RNAi fragment cassette, respectively.

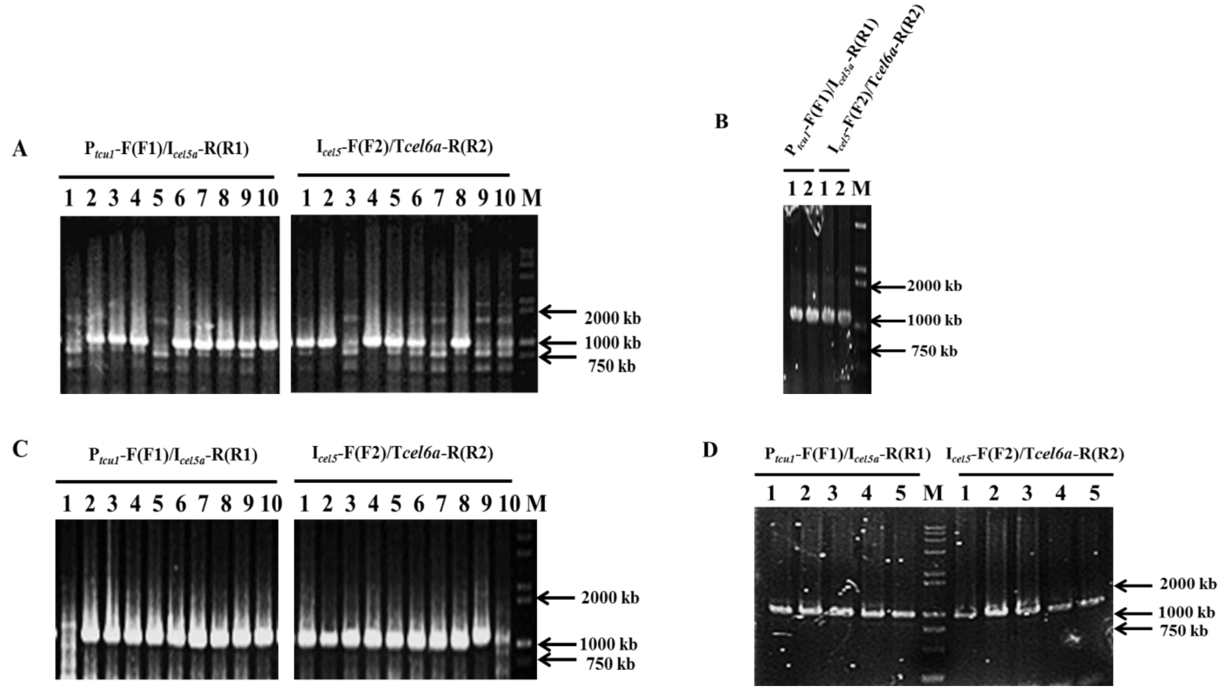

Figure S2 DNA gel electrophoresis of PCR products for verifying correct RNAi transformants. The PCR was performed by primer pairs P$_{tcu1}$-F(F1)/I$_{cel5a}$-R(R1) and I$_{cel5}$-F(F2)/T$_{cel6a}$-R(R2) using genome of P$_{tcu1}$-*pyr4*^{KD} (A), P$_{tcu1}$-*xyr1*^{KD} (B), P$_{tcu1}$-*cel7a*^{KD} (C) and P$_{tcu1}$-*fab1*^{KD} (D) as DNA templates.

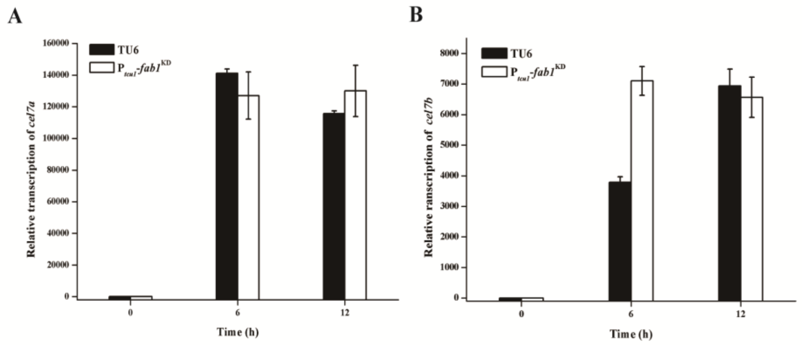

Figure S3 qRT-PCR analysis of the transcription of *cel7a* and *cel7b* in P_{tcu1}-*fab1*^{KD} and TU6 strains. The abundance of *cel7a* (A) and *cel7b* (B) mRNAs in P_{tcu1}-*fab1*^{KD} and TU6 strains under Avicel inducing condition without copper.

Table S1

Primers used in this study

Name	Sequence （5’-3’）
For plasmids construction	
P_{tcu1}-F	CCCAAGCTTCTGTGTGGCATCACTCAT
P_{tcu1}-R	CCGGATATCTGTCGTATCAACCAGGTCG
I_{cel5a}-F	CCGGATATCATCGGTACCGTGAGTACCCTTGTTTC
I_{cel5a}-R	GGGCGGCCGCACGACTAGTCTGTAACAAGACTTCCATTAA
T_{cel6a}-F	GGACTAGTGGCTTTCGTGACCGGGCTT
T_{cel6a}-R	ACGCGTCGACACGAGCTTGTGCTGCGGAATC
cel7a-F1	CCGGATATCCTTTACCTTATGGCGAGCGA
cel7a-R1	GGGGTACCGATTCGACCTGAGCAGGG
cel7a-F2	GGACTAGTGATTCGACCTGAGCAGGG
cel7a-R1	ATAAGAATGCGGCCGCCTTTACCTTATGGCGAGCGA
xyr1-F1	CCGATATCTTACCCGCTGGCAAATGG
xyr1-R1	GGGGTACCTGTTCAAGTCGTGCTCATCC
xyr1-F2	GGACTAGTTGTTCAAGTCGTGCTCATCC
xyr1-R2	ATAAGAATAGCGGCCGCTTACCCGCTGGCAAATGG
pyr4-F1	CCGATATCGTACCTGGCCGACAAGATTG
pyr4-R1	GGGGTACCGTTGAGCGTCTCCTGCGAGAT
pyr4-F2	GGACTAGTGTTGAGCGTCTCCTGCGAGAT
pyr4-R2	ATAAGAATGCGGCCGCGTACCTGGCCGACAAGATTG

fab1-F1	CCGATATCTCTCTTTCGACAGTCGAGACGT
fab1-R1	GGGGTACCAGATGCCAACAGTTTGGAGCTTGC
fab1-F2	GGACTAGTAGATGCCAACAGTTTGGAGCTTGC
fab1-R2	ATAAGAATAGCGGCCGCTCTCTTTCGACAGTCGAGACGT
For genomic PCR	
P_{tcu1}-F(F1)	CCACAAGAGCCTACTGCCAAATC
T_{cel6a}-R(R1)	TAGACAAAGACTCCGCCAACC
I_{cel5a} F(F2)	GTGAGTACCCTTGTTTCCTGGT
I_{cel5a}-R(R2)	CTGTAACAAGACTTCCATTAATTC
For qRT-PCR	
Q*cel7a*-F	CTTGGCAACGAGTTCTCTT
Q*cel7a*-R	TGTTGGTGGGATACTTGCT
Q*xyr1*-F	CCATCAACCTTCTAGACGAC
Q*xyr1*-R	AACCCTGCAGGAGATAGAC
Q*cel7b*-F	CGGCTACAAAAGCTACTACG
Q*cel7b*-R	CTGGTACTTGCGGGTGAT
Q*cel3a*-F	AGTGACAGCTTCAGCGAG
Q*cel3a*-R	GGAGAGGCGTGAGTAGTTG
Q*tcu1*-F	GCTGGTGGAAGAACTCAAGGA
Q*tcu1*-R	GCATGACTATGTACGCGGCTC
Q*fab1*-F	AGACGCTGGATGATCGGCTCGT
Q*fab1* -R	GAGGGGACTCGTAAATGTACT
